# Notes and Notices

**Published:** 1895-11

**Authors:** 


### NOTES AND NOTICES.

CORRECTION: In the October number, at page 462, a very serious error occurs. All that portion of the page, beginning with paragraph dated May 9th and ending with the one dated June 16th, both included, should be placed at the end of the article on page 467.

DR. THOMAS M. DILLINGHAM has removed his office from 46 West Thirty-sixth Street, to his residence, 8 West Fourty-ninth Street, New York City. Office hours: Dr. Dillingham, 10 A. M. to 12 M., 5 to 6 P. M.; Sundays, 12 to 1 P. M. Dr. Arthur G. Allan, 8 West Forty-ninth Street, 9 to 10 A. M., 2 to 5 P. M.; Sundays, 9 to 10.30 A. M., 5 to 6 P. M. Dr. Allan's residence, Hotel Imperial.

DR. H. P. MERA has removed his office and residence to 55 Adelaide Street, Detroit, Michigan.

DR. HENRI G. IDE, formerly of Cincinnati, Ohio, has purchased the practice of Dr. W. M. Wemp, of Oxford, Michigan, and will hereafter continue as the homœopathic physician of that thriving town.

DRS. MACLACHLAN & BROOKS desire to announce to their friends and patrons that they have formed a copartnership after having been associated in college and hospital work during the past year. Dr. MacLachlan will spend three days of each week in Ann Arbor. Dr. Brooks will reside in Ann Arbor, his residence being 31 East Jefferson Street, where he may be found at all times outside of office hours. He will engage also in the general practice of his profession, and will be in readiness to respond promptly to professional calls, both day and night.

They will retain their present office, corner Main and Washington Streets, Ann Arbor, Michigan, where both may be consulted.

Dr. D. A. MacLachlan, who has so long held the chair of Ophthalmology, Otology, and Laryngology, in the Homœopathic Medical Department of the University of Michigan, has removed from Ann Arbor to Detroit, and will hold himself in readiness to go to any part of the country, in response to professional calls, for either consultation or operation. Office: No. 6 Adams Avenue West, Grand Circus Park. Hours: 10 to 12 m. and 1.30 to 4 p. m.; telephone 3659, three rings; residence: 111 Edmund Place. Practice limited to diseases of the eye, ear, nose, and throat.

For Sale.—Volumes VIII, IX, XII, XIII, and XIV of The Homœopathic Physician, unbound, for sale. Inquire of Dr. Wm. Steinrauf, St. Charles, Mo. Box 146.

Hering College, of Chicago, opened October 1st, with music, refreshments, and enthusiastic speech-making. The reorganized Faculty and the students seemed eager for the new year's work.

The first number of the *Hering Bulletin* appeared at about the same time. This publication issues quarterly, and, while the initiatory number was edited by the Faculty, the students will have charge of it hereafter.

Whereas the medical clinics have heretofore taken the lead in Hering, the chairs of surgery have this year organized a series of clinical meetings that show the present and future prosperity and value of this department of surgical instruction. Cases have been various and abundant, and the students express great satisfaction on this account.

Well-wishers of pure Homœopathy will be glad to hear of the continued prosperity of this college. With its Faculty reorganized and strengthened future harmony is assured. The enthusiasm of its alumni and the interest of the profession in general are attested by the large number of applications for admission for the college year just begun. The phenomenal success of this school is based on a deep and wide-spread desire to see its great principles survive in their purity, and its influence in the homœopathic world is already marked.

The Homœopathic Medical College attached to the University of Michigan, at Ann Arbor, has been completely reorganized. A correspondent writes to us that the Faculty and students are entering upon the year's work with great interest. The commodious hospital has been put upon an entirely independent basis, as far as management is concerned, and the number of students is greatly increased. The whole class membership is made up of students who are pursuing their studies with an enthusiasm and loyalty that promise well for the cause.

